# The clinical effect of traditional chinese medicine on middle-aged women with Interstitial Cystitis: Protocol for a randomized controlled trial

**DOI:** 10.1097/MD.0000000000019673

**Published:** 2020-04-03

**Authors:** Yahong Liu, Pingan Zhang, Mengyu Liu, Xiaohe Liu, Ruijia Liu, Xudong Yu, Sheng Deng, Hongmei Si, Bei Sun

**Affiliations:** a The Second Affiliated Hospital of Shaanxi University of Chinese Medicine, Xianyang, Shaanxi, P.R. China; b Graduate School of Beijing University of Chinese Medicine; c Dongzhimen Hospital, Beijing University of Chinese Medicine, Beijng; d Chengde Medical University, Chengde, Hebei, China.

**Keywords:** Interstitial cystitis, randomized controlled trial, traditional chinese medicine

## Abstract

**Introduction:**

Interstitial cystitis (IC), as a common disease in urology, is prolonged and repeated. IC has caused great harm to the patient's physical and psychological. Traditional Chinese medicine (TCM) is characterized by overall concepts and dialectical treatment. It provides clinicians with safer and more reliable alternatives in terms of clinical prescriptions and prepared medicines, and also improves the quality of life of patients with IC. Therefore, in this study, we will use the research method of randomized controlled trials to explore the effects of TCM combined with western medicine on renal function and urine metabolism on middle-aged women with IC.

**Methods/design:**

Use randomized controlled trials. According to the proposed diagnostic, inclusion, and exclusion criteria. Sixty patients with interstitial bladder inflammation that met the criteria were randomized into a treatment group and a control group of 30 cases each. The intervention group was treated with integrated traditional Chinese and western medicine. The control group was given conventional Western medicine treatment. The course of treatment is 8 weeks. Interstitial bladder inflammation symptoms score (ICS worker), problem score (worker CPI), pelvic pain and urinary urgency symptoms, and urodynamics were used as the evaluation criteria.

**Discussion:**

This trial may provide evidence regarding the clinical effectiveness, safety, and cost-effectiveness of TCM for patients with IC.

**Trial registration:**

ClinicalTrials.gov, ChiCTR2000029971, Registered on 17 February 2020

## 1 Introduction

Interstitial cystitis (IC), as a common disease in urology, is prolonged and repeated. IC has caused great harm to the patient's physical and psychological. Because of its potential carcinogenicity, surgery must be used as a first-line treatment option.^[1]^ However, surgery and postoperative chemical drug treatment have not improved the quality of life of patients, and even brought great economic and physical and mental burden to patients.^[2]^ After analyzing the methods and experiences of domestic and foreign scholars in treating this disease in recent years, we believe that adding traditional Chinese medicine (TCM) decoction orally, infusion, or proprietary Chinese medicine treatment on the basis of conventional western medicine treatment can not only improve the clinical efficacy of patients, but also reduce surgery and western medicine.^[3]^ Adverse reactions caused by perfusion therapy. And can improve the clinical symptoms of patients. TCM is characterized by overall concepts and dialectical treatment. It provides clinicians with safer and more reliable alternatives in terms of clinical prescriptions and prepared medicines, and also improves the quality of life of patients with IC.^[4,5]^ Therefore, the use of Western medicine for eradication of diseased tissue. The use of ‘*Guben Quxie*’ therapy for the etiology and pathogenesis of integrated Chinese medicine can reduce costs and improve clinical efficacy. At present, the problem with integrated Chinese and western medicine in the treatment of glandular cystitis is the lack of systematic and theoretical large-scale randomized clinical trials of TCM, especially the lack of long-term efficacy observations, leading to the lack of evidence-based medical evidence, which limits clinical practice.^[6]^ Therefore, the focus of this study should be on the observation of the clinical efficacy of integrated Chinese and western medicine in the treatment of adenoid cystitis. Based on the concept of evidence-based medicine, multidisciplinary cooperation should be conducted to highlight the advantages of integrated Chinese and western medicine in the treatment of adenoid cystitis. Therefore, in this study, we will use the research method of randomized controlled trials to explore the effects of TCM combined with western medicine on renal function and urine metabolism on middle-aged women with IC. We hope that the results of this study will provide more evidence-based medical evidence for TCM to treat IC, and also provide patients with more treatment options.

## 2 Methods/design

### 2.1 Study design and settings

**Figure 1:**
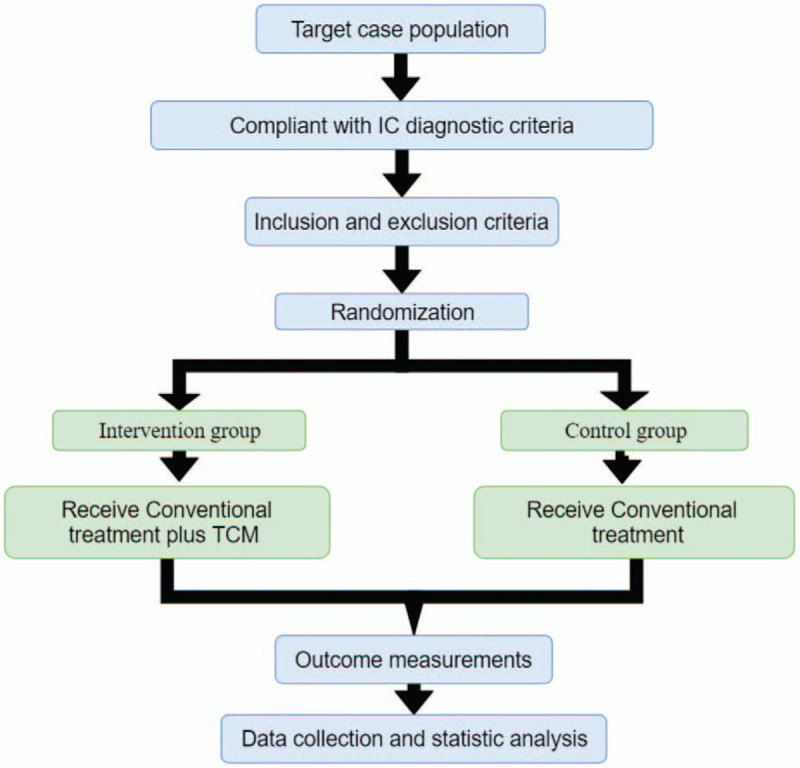
SPIRIT figure for the schedule of enrollment, interventions, and assessments. FDV = Initial volume at the first desire to void, ICPI = Interstitial Cystitis Problem Index, ICSI = Interstitial Cystitis Symptom Index, MCC = Maximum cystometric capacity, PUF = the pelvic pain and Urgency or frequency, SPIRIT = Standard Protocol Items: Recommendations for Interventional Trials, TCM = traditional Chinese medicine.

This study will be a single-blinded, randomized controlled trial with 2 parallel groups. It will be conducted at the The Second Affiliated Hospital of Shaanxi University of Chinese Medicine. This protocol was written and based on Standard Protocol Items: Recommendations for Interventional Trials guidelines The participants will be informed about the research, procedures, risks, and benefits by HGE (author of this protocol). If they agree, they will sign an informed consent form. Only those participants who read and agree to the protocol and who sign the informed consent form will take part of the study, following the schedule described in Figure 1.

### 2.2 Participants

**Figure 2:**
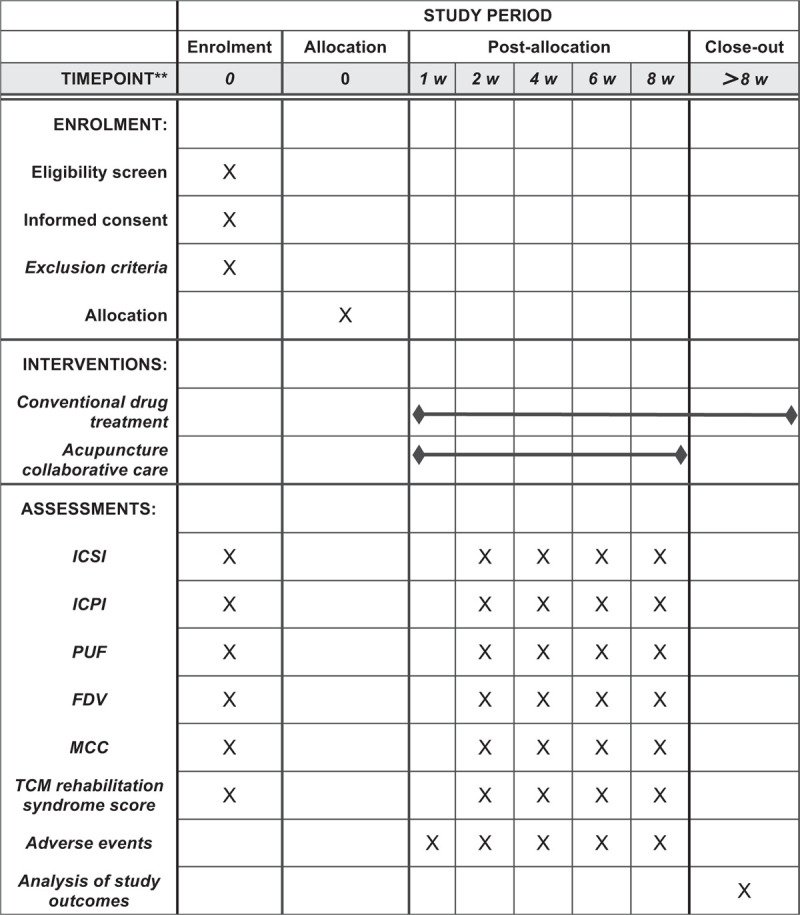
Study design flow chart.

The selected cases will be both outpatients and inpatients at The Second Affiliated Hospital of Shaanxi University of Chinese Medicine. The diagnostic criteria refer to the “Urological Surgery · Interstitial Bladder Inflammation Diagnostic Standard” (Wu Jieping, Gu Fangliu, Guo Yinglu, Yang Yong Wu Jieping, Beijing Shandong Science and Technology Press).(Fig. 2)

#### 2.2.1 Diagnostic criteria

Western medicine diagnosis refers to “Chinese Urological Disease Diagnosis and Treatment Guide” and “Urinary Surgery” in kidney stone related diagnostic standards. The diagnosis of TCM refers to the “Guiding Principles for Clinical Research of New Chinese Medicines,” and the syndrome is identified as damp-heat-stasis syndrome type.

#### 2.2.2 Inclusion criteria

This study will be conducted in China. Patients will be recruited from Urology/Andrology departments of The Second Affiliated Hospital of Shaanxi University of Chinese Medicine. We will enroll participants based on the following inclusion criteria:

(1) No urinary frequency, urgency, or pain in the bladder with a clear cause; urine culture was negative.
(2) Under anesthesia, spotted bleeding or ulcers can be seen on cystoscopy after water expansion. The formation of bleeding points should occur after the bladder is irrigated with an inflation pressure of 80 ∼ 100 cm H20 for 1 to 2 minutes. Cystoscopy should be performed after the bladder is inflated more than 2 times. The hemorrhage formation site is diffuse, and it exists in at least 3 quadrants of the upper body, and each quadrant has at least 10 lesions.
(3) Accept and sign the informed consent

#### 2.2.3 Exclusion criteria

Patients will be excluded if they meet the following criteria:

This study will be conducted in China. Patients will be recruited from Urology/Andrology departments of The Second Affiliated Hospital of Shaanxi University of Chinese Medicine. We will enroll participants based on the following inclusion criteria:

(1) The volume is greater than 350 mL when measuring bladder pressure;
(2) Perfusion is performed at a rate of 30 to 100 mL/min during bladder pressure measurement, and no urgency to urinate when 100 mL of gas or 150 mL of liquid is reached
(3) Periodic involuntary bladder contraction during perfusion at the above rate;
(4) Symptoms less than 9 months and no nocturia;
(5) Remission after treatment with antibiotics, cholinergic inhibitors or antispasmodics, muscle relaxants;
(6) Less than 8 urinations during the day when awake;
(7) Diagnosis of bacterial bladder inflammation within 3 months;
(8) Suffering from bladder or lower urinary tract stones;
(9) Suffering from active genital sores; uterine, cervical and vaginal urethral tumors;
(10) Tuberculous cystitis;
(11) Radiation cystitis or benign and malignant bladder tumors;

#### 2.2.4 Conditions for participants to suspend and withdraw from the clinical trial

Researchers participating in clinical trials should carefully record the reasons for the suspension of the trial and the relationship with the clinical trial. It is necessary to clearly record the unwillingness of the subjects to continue the clinical trials, put forward the reasons for withdrawing from the clinical trials, and record the evaluation indicators at the time of discontinuation in detail.

(1) Those who cannot adhere to treatment;
(2) Allergic reactions or serious adverse reactions during the test, the test should be suspended:
(3) Those who have not been treated strictly according to the plan;
(4) People who withdrew from the study on their own

### 2.3 Interventions

Control group: Participants in this group will be given oral imipramine twice daily at 50 mg each; heparin 100 mL intravesical perfusion once a week. The course of treatment is 8 weeks.

Intervention group: This group will be given the TCM prescription-Longdan Xiegan Decoction, 1 dose daily, one morning and one evening. The composition of TCM prescription is: Longdancao 15 g, Caihu 15 g, Zhizi 10 g, Huangqin 15 g, Shengdi 15 g, Cheqianzi 15 g, Zexie 15 g, Danggui 5 g, Mutong10 g, Niuxi10 g, Gancao5 g. At the same time, patients in this group received conventional intravesical infusion. The treatment of intravesical perfusion is the same as that of the control group.

The total application time will be 8 weeks. At the same time, closely monitor the change of the condition in order to control the deterioration of the condition in time.

### 2.4 Outcome measures

Baseline and postintervention outcome variables and potential confounders will be measured in both the intervention and control groups. Measurements will be performed before surgery, after 6 weeks, and the end of the program (8 weeks).

#### 2.4.1 Primary outcome measures

We will use the O ’Leary Saint Interstitial Cystitis Symptom Index, the 0’ Leary Saint IC Problem Index, and the pelvic pain and Urgency or frequency symptom score was used as the main outcome measures.

#### 2.4.2 Secondary outcome measures

We will detect changes in bladder volume through urodynamics. A urodynamic tester produced by Laborie of Canada was used. Urodynamic monitoring indicators:

(1) Initial volume at the first desire to void;
(2) Maximum cystometric capacity;
(3) Maximum urine flow rate (Qmax).

#### 2.4.3 Efficacy evaluation

Refer to “Guidelines for Clinical Research of New Chinese Medicine.” Efficacy was evaluated by the ratio of the difference between the points before and after treatment compared to the points before treatment. Syndrome treatment efficiency = (total points before treatment total points after treatment)/total points before treatment × 100%.

(1) Clinical control: clinical symptoms and signs disappeared or basically disappeared, and syndrome scores were reduced by ≥95%;
(2) Significant effect: clinical symptoms and signs were significantly improved, and syndrome scores were reduced by 70%;
(3) The signs and symptoms have improved, and the syndrome scores have decreased by ≥30%;
(4) Ineffective: the clinical symptoms and signs have not improved significantly, and the syndrome scores have decreased by less than 30%.

### 2.5 Sample size calculation

The sample size for this trial is based on an expected mean difference between groups of 11 points of the Constant–Murley questionnaire, which is the minimum clinically important difference. The mean assumed for the calculation was 63.3 with a standard deviation of 15 points based on results of other randomized clinical trials. To detect this difference between both treatments, with a value of a = 0.05 (probability of committing a type I error) and a statistical power of 95%, a minimum of 49 patients per group is needed. This minimal sample size estimate has been increased by 20% after considering the potential dropouts, finally including 60 patients for each group. Accordingly, the proposed experimental hypothesis is that there will be a difference of at least 11 points in the Constant—Murley questionnaire in the intervention group versus the control group. The sample size was determined using the Stata SE software, version 15.

### 2.6 Randomization and blinding

Participants will be randomly allocated to the 2 groups through a sequence of numbers generated by a computer program before starting the selection process. The group assigned to each patient will be kept in a sealed envelope with the objective of concealing the assignment to the researcher, who will decide on the entry of subjects to the study. Given the nature of the interventions, the physiotherapists, and the patients, blinding will not be possible. However, the evaluator and statistician will be blinded to which group the subjects evaluated will belong.

### 2.7 Statistical analysis

Data management uses EXCEL software to build a database, double entry, check for outstanding values, and lock. Statistical analysis will be performed using SPSS 25.0 software for statistical analysis. The normality of the measurement data is tested. The data obeying the normal distribution is Student *t* test, which is expressed by mean ± standard deviation. The data not obeying the normal distribution is rank sum test. And marginal homogeneity test; count data are expressed by rate and composition ratio, and comparison is performed by chi-square test; repeated measurement data are expressed by mean ± standard deviation, intra-group comparison is performed by analysis of variance of repeated measurement data, and inter-group comparison is by multivariate analysis of variance. *P*≤.05 indicates that the difference is statistically significant.

### 2.8 Data management

Information obtained from the evaluation of each participant will be recorded on a paper print-out. The information will then be handwritten on a paper document case report form and entered into an Excel file for future statistical analyses. In accordance with the Personal Information Protection Act, the names of all participants will not be disclosed, and a unique identifier number given during the trial will be used to identify participants. All of the participants will be informed that the clinical data obtained in the trial will be stored in a computer and will be handled with confidentiality. The participants’ written consent will be stored by the principal investigator.

### 2.9 Ethics

The study will be conducted under the Declaration of Helsinki principles, as well as following the norms of good clinical practice. Recruitment of patients has not started in this study. The study plan will be submitted to the ethics committee of the Second Affiliated Hospital of Shaanxi University of TCM for review. The study protocol will be approved by the ethics committee of the Second Affiliated Hospital of Shaanxi University of TCM. The protocol of this study has been registered in the Chinese Clinical Trial Registry with the number ChiCTR2000029971.

## 3 Discussion

IC is a chronic urinary system disease. The number of patients with this disease is large, and the treatment effect is poor. It is more common in middle-aged women aged 30 to 50 years. The incidence rate is about 3/1000-51/1000, and the incidence rate is increasing year by year.^[7]^ There is no exact census statistics for this disease. However, more and more literature reports suggest that the disease is widespread in the population. IC has a high prevalence and a long course of disease, and its condition is repetitive and chronically progressive.^[8,9]^ As the disease progresses, most patients experience continuous deterioration in their physical health. At the same time, due to the expenditure on diagnosis and treatment of the disease, the patient's financial burden increases, while the economic income declines, and the family status and social status decrease.^[10]^ Under such circumstances, patients are prone to emotional disorders such as inferiority, depression, depression, and anxiety. Therefore, to comprehensively evaluate the survival status of IC patients, it is not comprehensive to rely solely on traditional indicators such as survival time, urine analysis, signs, imaging, liver and kidney function, and urodynamic tests.^[11]^ By the same token, the treatment of chronic diseases such as IC should aim at reducing the impact of the disease on patients’ daily lives and improving the quality of life and physical and mental health of patients. It cannot be based on the improvement of physiological indicators.^[12,13]^ In contrast, the use of symptom assessment as an indicator has more comprehensive and reasonable characteristics, and can better reflect the patient's psychophysiological needs, so it has important clinical and social significance.

How to prevent and improve the quality of life of IC patients and find an effective and easy-to-receive method has become an issue of increasing concern in the medical community. The prevalence of IC is high, the disease duration is long, and the patient's viability and quality of life are reduced.^[14]^ The condition is recurrent and shows a chronic progressive development process. So far, IC has no specific treatment. Therefore, while seeking effective methods for the treatment of IC, strengthen the clinical intervention of patients in stable phase. Relieving clinical symptoms, improving patients’ quality of life, and improving prognosis are the hot spots in the treatment of IC today. TCM has a unique overall concept and rich practical experience, and is safe to use with fewer adverse reactions and side effects. A large number of clinical studies have proven that medicine has unique advantages in the treatment of urological diseases.^[15]^ Therefore, the use of TCM to intervene patients has been increasingly valued by the medical community.

## Acknowledgments

The authors would like to thank all the trial participants. The authors are grateful for the support for this study: trial coordinating team, surgical staff, nurses, and research departments.

## Author contributions

YHL, XDY, BS, PAZ, and RJL designed the study protocol and drafted the manuscript. HXL and MYL reviewed the study protocol and drafted the manuscript. SD and YHL is responsible for the statistical design and analysis as trial statistician. All authors carefully read and approved the final version of the manuscript.
